# Comparison of Structured and Conventional Viva Voce in Pharmacology Teaching Among Second-Year Bachelor of Medicine, Bachelor of Surgery (MBBS) Students: A Prospective Study

**DOI:** 10.7759/cureus.109231

**Published:** 2026-05-19

**Authors:** Mohit Kulmi, Sameer Pandit, Pallav Hetawal, Devendra Pandya, Somdutt Rawat

**Affiliations:** 1 Pharmacology, Dr. Laxmi Narayan Pandey Government Medical College, Ratlam, IND

**Keywords:** faculty members, medical education assessment, medical school students, pharmacology learning, viva voce

## Abstract

Background

Traditional viva voce has long been a cornerstone of student evaluation in medical education. Despite its widespread use, it is often criticized for subjectivity, variability, and lack of standardization. This study explored the feasibility of structured viva voce as an alternative assessment tool in pharmacology.

Methods

A prospective educational interventional study was conducted among 83 second-year Bachelor of Medicine, Bachelor of Surgery (MBBS) students as part of the Advanced Course in Medical Education (ACME) faculty development program. Each student was assessed using both traditional and structured viva formats. Scores were analyzed quantitatively, and perceptions of students and faculty were collected using a validated questionnaire comprising Likert-scale items and open-ended questions.

Results

The mean scores obtained in the structured viva were significantly higher compared to the traditional format (5.36 vs. 4.64; p=0.0242). Feedback analysis demonstrated that both students and faculty expressed predominantly favorable perceptions of the structured viva, citing improved fairness, clarity, and comprehensiveness in knowledge assessment.

Conclusion

Structured viva voce is a reliable and effective assessment modality in pharmacology education. Its implementation can enhance objectivity and improve the evaluation process compared to traditional viva voce examinations.

## Introduction

Pharmacology is a fundamental discipline in medical education that requires students to acquire, integrate, and apply knowledge for rational therapeutic decision-making in clinical practice [1,2]. Effective assessment methods are essential not only for evaluating student performance but also for guiding learning and improving teaching strategies. Viva voce examinations are a core component of medical assessment, enabling the evaluation of higher-order cognitive skills, communication abilities, and professional attitudes [3]. However, the traditional viva format is often criticized for subjectivity, low reliability, inter-examiner variability, lack of standardization, and potential bias [4]. A recent systematic review and meta-analysis by Abuzied and Nabag reported that traditional viva demonstrates relatively low reliability (Cronbach's alpha: ~0.50), whereas structured viva formats achieve higher reliability (0.75-0.80) and improved learner acceptability [5].

Structured viva voce, characterized by standardized questions, predefined scoring schemes, and uniform administration, has been proposed as a more objective alternative. Previous studies have demonstrated improved validity, reliability, and student satisfaction with structured formats [5,6]. Ahsan and Mallick reported a significant correlation between structured viva scores and theory examination performance, supporting its concurrent validity [7]. Similarly, Loomba and Jindal demonstrated improved objectivity and stakeholder acceptance following the introduction of structured oral examinations in undergraduate biochemistry [8]. Additionally, prior exposure to structured formats has been shown to reduce student anxiety and enhance performance [6].

Despite these advantages, structured viva requires careful planning, faculty training, and resource allocation. Moreover, its feasibility and effectiveness may vary across institutional contexts. Therefore, this study was undertaken to compare structured and conventional viva voce in pharmacology among second-year Bachelor of Medicine, Bachelor of Surgery (MBBS) students, with the objective of evaluating performance outcomes and stakeholder perceptions.

## Materials and methods

Study design and setting

This prospective educational interventional study was conducted in the Department of Pharmacology at Dr. Laxmi Narayan Pandey Government Medical College, Ratlam, India, from January to February 2022. The study was conducted as part of the 11th Batch of the Advanced Course in Medical Education (ACME), a National Medical Commission (NMC) faculty development program, in India, focused on improving teaching-learning and assessment practices in medical education. The program included training sessions on educational objectives, assessment methods, structured evaluation techniques, and implementation of competency-based teaching approaches. As part of the course activities, participants were encouraged to design and implement educational interventions within their departments. In the present study, faculty members received orientation regarding the structured viva format, scoring system, and standardized assessment procedures prior to the conduct of examinations. Both traditional and structured viva examinations were conducted under uniform conditions following predefined protocols to ensure consistency in assessment.

Ethical considerations

Ethical approval was obtained from the Institutional Ethics Committee of Dr. Laxmi Narayan Pandey Government Medical College with approval number GMC RATLAM/2021/IEC/Approval/12, dated 13/12/2021, prior to the commencement of the study. A waiver of written informed consent was granted by the Institutional Ethics Committee because the study constituted a minimal-risk educational intervention integrated into routine academic activities. Student participation was voluntary, anonymity and confidentiality were ensured, and no modification of standard teaching or assessment practices adversely affected participants.

Participants

The study population comprised second-year MBBS students (batch 2019). The sample size was calculated based on a total population of 180 second-year MBBS students using a 95% confidence level and a 5% margin of error, assuming maximum variability (response distribution of 50%). The estimated minimum sample size obtained was 123; however, due to feasibility constraints and voluntary participation, 83 students were ultimately included in the study. As this was an educational interventional study with paired assessment of the same participants using both traditional and structured viva formats, each student served as their own control, which improved the study's ability to detect differences between assessment methods.

Study instruments

A structured viva voce questionnaire was developed covering the pharmacology syllabus. Eight question cards were prepared, each containing 16 questions arranged in increasing order of difficulty. A predefined scoring system was used with a total score of 10 marks, comprising 10 easy questions carrying 0.5 marks each, four moderate questions carrying 0.75 marks each, and two difficult questions carrying 1 mark each. The questionnaire and scoring scheme were validated by departmental faculty. Separate validated feedback questionnaires were used for students (13 items) and faculty (eight items), incorporating Likert-scale responses and open-ended questions.

Data collection procedure

Participants were oriented to the study protocol prior to assessment. All viva examinations were conducted in standardized examination room settings with similar environmental conditions to ensure consistency across assessments.

Traditional Viva Voce Administration

Each traditional viva was conducted by a single faculty examiner following the conventional unstructured format. Students were asked questions spontaneously based on the pharmacology curriculum without a predetermined question set. The duration was approximately 5-7 minutes per student. Examiners assessed students on a 10-point scale based on their subjective evaluation of overall performance, knowledge depth, and communication skills. No standardized scoring rubric was used.

Structured Viva Voce Administration

Each structured viva was conducted by a different faculty examiner to minimize bias. Students were allocated one of eight pre-validated question cards through random selection at the time of assessment. Each card contained 16 questions arranged in hierarchical difficulty: 10 easy questions (0.5 marks each), four moderate questions (0.75 marks each), and two difficult questions (1 mark each), totaling 10 marks. The duration was approximately 5-7 minutes per student. Questions were asked sequentially in the predetermined order. Examiners had standardized answer keys available during the assessment and assigned marks at the end of the viva according to the predefined marking scheme.

Examiner Standardization

A total of six faculty examiners from the Department of Pharmacology participated in the study. All examiners underwent a standardization and training session on the structured viva format, scoring scheme, and assessment procedures prior to conducting the examinations. To minimize bias, no examiner assessed the same student in both formats, and examiners were blinded to the students' scores in the alternate assessment format.

Temporal Arrangement and Randomization

Both assessments were completed within a two-week period. The sequence of assessment (traditional first vs. structured first) was randomized across students to control for order effects. The two viva formats were conducted seven days apart to minimize fatigue and learning effects while ensuring comparable baseline knowledge. For administrative convenience, students were divided into four groups (Groups A-D) based on routine departmental batch allocation, with assessments conducted over four days (one group per day). However, this scheduling arrangement did not affect the time interval between the two viva formats for individual students.

Environmental Standardization

All vivas were conducted in similar examination rooms under standardized conditions to ensure a quiet, interruption-free environment conducive to fair assessment. Feedback questionnaires were collected from students immediately after the completion of both assessments.

Statistical analysis

Data were entered into Microsoft Excel (Microsoft Corporation, Redmond, Washington, United States) and analyzed using appropriate statistical methods. Quantitative data were expressed as mean and standard deviation, while qualitative data were presented as frequencies and percentages. A paired Student's t-test was used to compare mean scores between structured and traditional viva formats. Effect size was calculated using Cohen's d to assess the practical significance of the difference. A p-value of <0.05 was considered statistically significant. Qualitative data from open-ended questions were analyzed using thematic analysis. Responses were independently reviewed, coded, and categorized into emerging themes related to perceived advantages, disadvantages, and suggestions for improvement of the structured viva format. Themes were identified based on the frequency and relevance of student responses.

## Results

**Figure 1:**
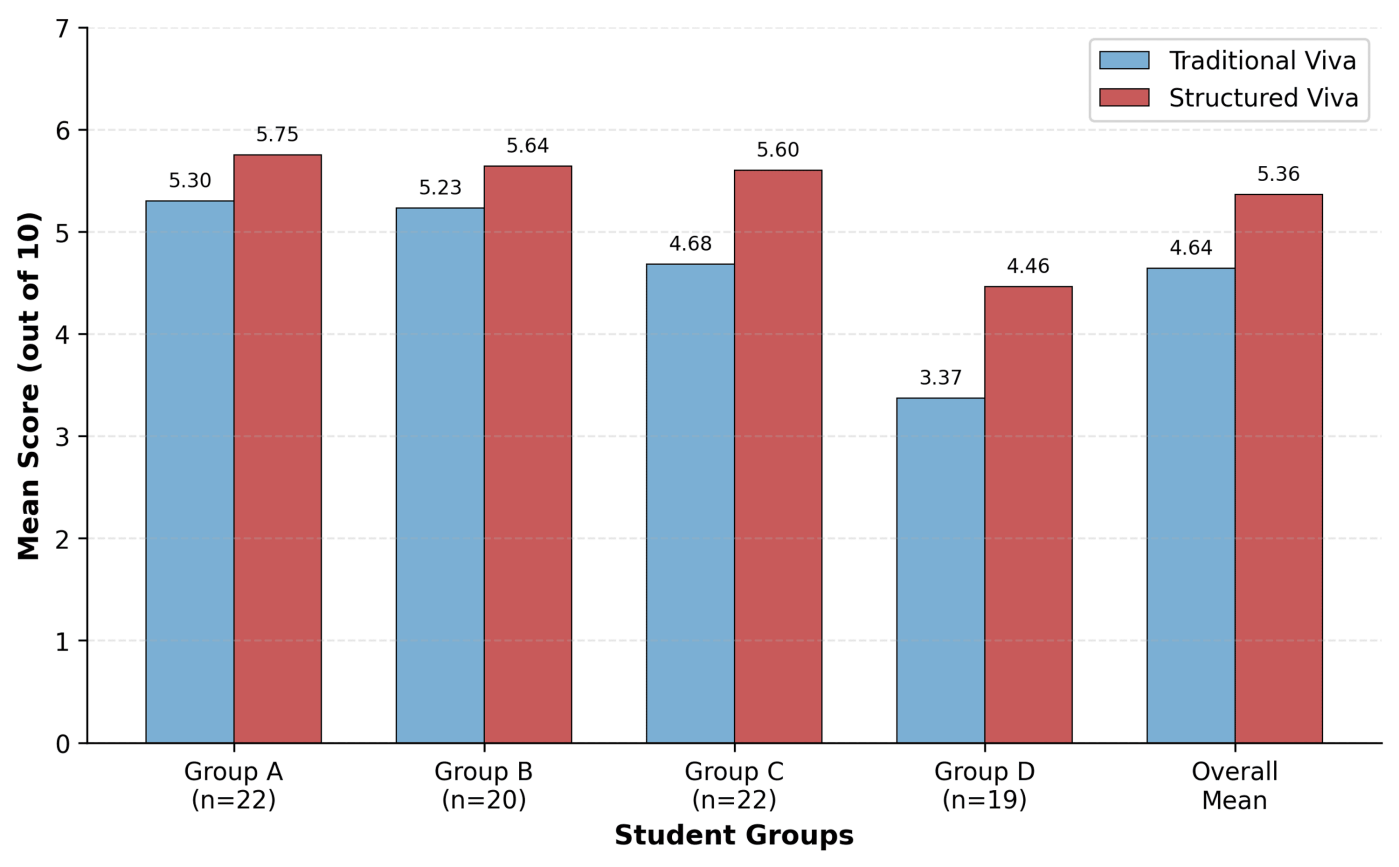
Group-wise comparison of mean scores in traditional and structured viva voce Bar graph showing the mean scores of students across different groups (A-D) and the overall mean in traditional and structured viva formats. Structured viva consistently yielded higher mean scores across all groups, indicating improved performance compared to the traditional method.

A total of 83 second-year MBBS students participated in the study. All students underwent both traditional and structured viva voce assessments. The mean score obtained in the structured viva (5.36) was higher than that in the traditional viva (4.64). This difference was statistically significant (p=0.0242) using a paired Student's t-test, with a medium effect size (Cohen's d=0.46), indicating improved student performance with the structured format. A detailed description is shown in Figure 1.

**Figure 2:**
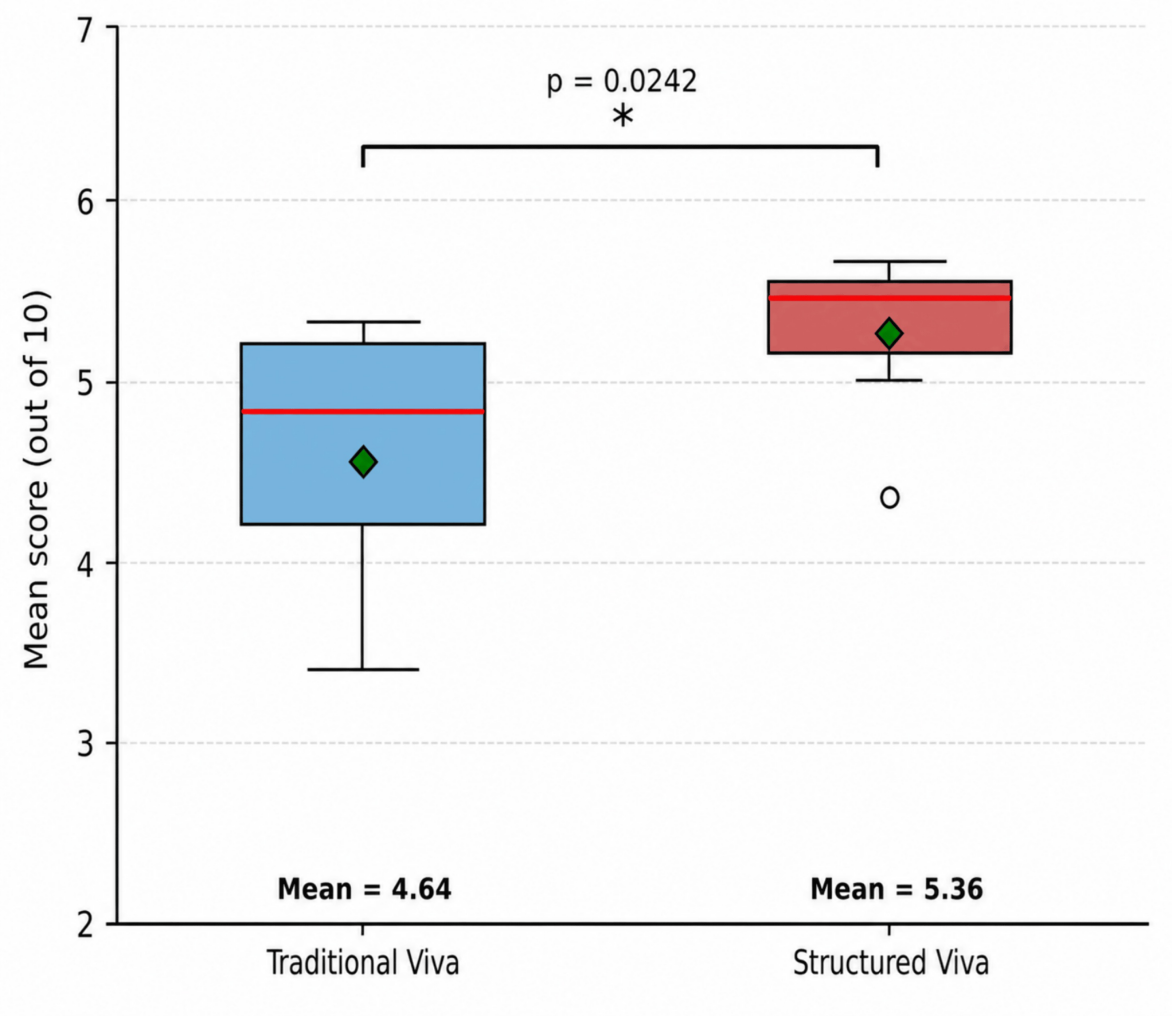
Mean difference in scores between structured and traditional viva Paired Student's t-test was used to compare the mean scores between structured and traditional viva formats. * indicates a statistically significant difference between the two assessment formats (p<0.05) using paired Student's t-test.

The difference in scores between structured and traditional viva was further analyzed using a paired Student's t-test. The mean difference (structured−traditional) was +0.72 points, indicating higher scores in the structured format. The 95% confidence interval ranged from 0.18 to 1.26 and did not cross zero, confirming statistical significance. The paired t-test demonstrated a significant difference between the two assessment methods (t(3)=4.23; p=0.024). The effect size (Cohen's d=0.46) indicated a medium practical significance of this difference. These findings indicate that structured viva is associated with a statistically significant and clinically meaningful improvement in student performance compared to the traditional format. The findings are illustrated in Figure 2

Student feedback

Student feedback was obtained using a 13-item questionnaire comprising 10 Likert-scale items and three open-ended questions. Overall, the majority of students (>75% combined "agree" and "strongly agree" responses across all items) expressed positive perceptions of the structured viva format. Specifically, 97.2% of students agreed or strongly agreed that structured viva was more organized, 87.2% found it more transparent, and 78.6% perceived it as free from bias compared to traditional viva. Regarding assessment effectiveness, 75.7% agreed that structured viva was an effective assessment tool, and 81.4% believed it would improve their performance in final examinations.

However, a small minority expressed disagreement or neutral opinions on certain aspects. Regarding question difficulty distribution, 11.4% disagreed that questions covered all difficulty levels, while 18.6% remained neutral. Time adequacy was contested by 10% of respondents, with an additional 12.9% expressing neutral views. Topic coverage received disagreement from 10% of students. Notably, 22.9% remained neutral about the effectiveness of structured viva as an assessment tool, suggesting uncertainty among some students despite overall positive trends. The detailed responses are shown in Table 1.

**Table 1:** Student feedback on structured viva voce using Likert-scale responses (n=70) Distribution of responses to Likert-scale items evaluating the organization, clarity, transparency, fairness, topic coverage, and overall effectiveness of structured viva. Responses were recorded on a five-point Likert scale ranging from strongly disagree to strongly agree.

Question	Strongly disagree	Disagree	Neutral	Agree	Strongly agree
Do you think structured viva is a more organized system?	0	1 (1.4%)	1 (1.4%)	44 (62.9%)	24 (34.3%)
Do you think that the questions were from all difficulty levels?	1 (1.4%)	7 (10%)	13 (18.6%)	30 (42.9%)	19 (27.1%)
Do you think that the time allotted was adequate for the viva?	1 (1.4%)	6 (8.6%)	9 (12.9%)	39 (55.7%)	15 (21.4%)
Do you think this is a more transparent process compared to traditional viva?	0	1 (1.4%)	8 (11.4%)	37 (52.9%)	24 (34.3%)
Do you think structured viva is more beneficial in improving performance in the final examination?	0	2 (2.9%)	11 (15.7%)	29 (41.4%)	28 (40%)
Were the questions easy to understand compared to traditional viva?	0	3 (4.3%)	10 (14.3%)	34 (48.6%)	23 (32.9%)
Do you think structured viva is an effective tool for assessment compared to traditional viva?	0	1 (1.4%)	16 (22.9%)	32 (45.7%)	21 (30%)
Do you think all the topics were equally covered in the structured viva?	0	7 (10%)	8 (11.4%)	40 (57.1%)	15 (21.4%)
Do you think structured viva is free from biases compared to traditional viva?	0	0	15 (21.4%)	34 (48.6%)	21 (30%)
Do you think the atmosphere of structured viva was comfortable compared to traditional viva?	1 (1.4%)	4 (5.7%)	5 (7.1%)	39 (55.7%)	21 (30%)

Qualitative student feedback

Qualitative feedback from students was analyzed using responses to open-ended questions, focusing on perceived advantages, disadvantages, and suggested improvements of the structured viva format. A summary of the thematic analysis conducted under various themes is given in Table 2.

**Table 2:** Student responses to open-ended questions on structured viva voce (n=70) Summary of qualitative feedback from students regarding the advantages, disadvantages, and suggested improvements of structured viva compared to traditional viva.

Question	Responses
What are the advantages of structured viva compared to traditional viva?	Difficulty level gradually increases, making it easier to answer. Questions cover all topics systematically. Hierarchical progression helps in assessing knowledge.
What are the disadvantages of structured viva?	Depth of knowledge cannot be tested adequately. Less time is allotted. Limited opportunity to explore a topic in detail. No hints from examiners.
What changes would you suggest?	Increase time allotted. Include two to three examiners. Avoid strict arrangement based on difficulty level.

Faculty feedback

Faculty perceptions of the structured viva were assessed using a Likert-scale questionnaire (n=6 faculty members). The faculty responses demonstrated predominantly positive attitudes toward structured viva. All six faculty members (100%) agreed or strongly agreed that structured viva was more reliable, helped students perform better, and reduced bias compared to traditional viva. Similarly, 100% supported its implementation as a standard assessment method in university examinations. Regarding time efficiency, one faculty member (16.7%) expressed a neutral opinion about whether structured viva consumed less time compared to traditional viva, while the remaining 83.3% agreed or strongly agreed. Two faculty members (33.3%) reported neutral comfort levels with conducting examinations through structured viva, while 50% agreed and 16.7% strongly agreed, suggesting that complete comfort with the new format may require additional experience or training. Notably, one faculty member (16.7%) disagreed that structured viva was free from bias, despite the majority (83.3%) agreeing or strongly agreeing with this statement. This isolated disagreement highlights the importance of continued examiner training and standardization to address potential concerns about objectivity. The responses are presented in Table 3.

**Table 3:** Faculty feedback on structured viva voce using Likert-scale responses (n=6) Values are presented as number (percentage) of responses. Responses were recorded on a five-point Likert scale ranging from strongly disagree to strongly agree.

Question	Strongly disagree	Disagree	Neutral	Agree	Strongly agree
Do you think structured viva is free from biases compared to traditional viva?	0	1 (16.7%)	0	3 (50%)	2 (33.3%)
Do you think structured viva helps students perform better?	0	0	0	3 (50%)	3 (50%)
Do you think assessment via structured viva is more reliable compared to traditional viva?	0	0	0	3 (50%)	3 (50%)
Do you think structured viva consumes less time compared to traditional viva?	0	0	1 (16.7%)	4 (66.7%)	1 (16.7%)
Do you think structured viva should be implemented in university examinations as standard?	0	0	0	4 (66.7%)	2 (33.3%)
Are you comfortable conducting examinations through structured viva?	0	0	2 (33.3%)	3 (50%)	1 (16.7%)

## Discussion

Medical education is defined as a lifetime learning process stretching from undergraduate to postgraduate and specialty training and beyond [9]. Viva voce examinations play a crucial role in medical assessment by enabling the evaluation of higher-order cognitive skills, communication, and clinical reasoning [3]. However, traditional viva formats are often limited by subjectivity, inter-examiner variability, and lack of standardization [4]. Structured viva voce has been shown to provide a more equitable and consistent evaluation, with higher reliability and learner acceptability compared to traditional methods [5,10].

In the present study, students performed significantly better in the structured viva compared to the traditional format (mean 5.36 vs. 4.64; p=0.0242). Similar findings have been reported in previous studies, where structured oral examinations were perceived as fair and effective tools for assessing knowledge and clinical reasoning [7,8]. Additionally, structured assessments have been widely regarded as valid and acceptable methods of formative evaluation by both students and examiners [11]. The positive student perceptions observed in this study, particularly regarding organization, transparency, and reduced bias, are consistent with findings from earlier research [3,5,12].

Despite these advantages, some students reported limited opportunities for in-depth exploration and insufficient time allocation. This reflects a known limitation of structured formats, where breadth of assessment may be prioritized over depth. Previous studies have similarly noted that structured viva improves coverage and feasibility but may restrict detailed probing of specific topics [13]. Hybrid approaches combining structured and traditional elements have been suggested as a potential solution, offering a balance between objectivity and flexibility [6,14].

Faculty feedback in the present study was also favorable, with most respondents supporting structured viva as a more reliable and less subjective assessment method. Similar observations have been reported in the literature, where structured oral examinations have been recognized as feasible and effective tools for improving standardization and assessment quality [8]. The positive reception in this study may also be attributed to prior orientation and standardized question design, highlighting the importance of implementation strategies alongside assessment format.

Limitations

This study has several limitations. First, it was conducted at a single institution with a relatively small sample size, which may limit the generalizability of the findings. Second, inter-rater reliability between examiners was not assessed, which could influence the objectivity of both traditional and structured viva formats. Third, the study evaluated immediate performance outcomes without assessing the long-term retention of knowledge or correlation with summative examination results. Additionally, the faculty sample size was small, which may affect the robustness of faculty perception data. Finally, although efforts were made to standardize the structured viva, factors such as examiner variability and time constraints could still have influenced student performance.

Future directions

Future studies should include multi-center designs with larger and more diverse student populations to enhance external validity. Assessment of inter-rater reliability and incorporation of standardized examiner training modules would further strengthen the evaluation process. Longitudinal studies examining the impact of structured viva on knowledge retention, clinical reasoning, and performance in summative examinations are warranted. Additionally, exploring hybrid models that combine structured frameworks with opportunities for in-depth discussion may help balance objectivity with depth of assessment. The integration of digital tools and automated scoring systems may also improve standardization and feasibility in large-scale implementations.

## Conclusions

Structured viva voce is a reliable, objective, and well-accepted assessment method in pharmacology education. It significantly improves student performance while enhancing fairness, transparency, and standardization compared to traditional viva formats. Despite certain limitations, particularly regarding depth of assessment and time constraints, structured viva demonstrates strong potential for integration into undergraduate medical curricula. Adoption of structured or hybrid viva models, supported by faculty training and institutional planning, can contribute to more consistent and effective assessment practices in medical education.
